# A genetic fingerprint of Amphipoda from Icelandic waters – the baseline for further biodiversity and biogeography studies

**DOI:** 10.3897/zookeys.731.19931

**Published:** 2018-01-23

**Authors:** Anna M. Jażdżewska, Laure Corbari, Amy Driskell, Inmaculada Frutos, Charlotte Havermans, Ed Hendrycks, Lauren Hughes, Anne-Nina Lörz, Bente Stransky, Anne Helene S. Tandberg, Wim Vader, Saskia Brix

**Affiliations:** 1 Laboratory of Polar Biology and Oceanobiology, Department of Invertebrate Zoology and Hydrobiology, Faculty of Biology and Environmental Protection, University of Lodz, 12/16 Banacha st., 90-237 Lodz, Poland; 2 Muséum National d’Histoire Naturelle MNHN, UMR7205 ISyEB, 43, rue Cuvier, CP 26, 75005 Paris, France; 3 Smithsonian Institution, Laboratories of Analytical Biology, National Museum of Natural History, Smithsonian Institution, Washington DC, USA; 4 University of Hamburg, Centre of Natural History, Zoological Museum, Martin-Luther-King-Platz 3, 20146 Hamburg, Germany; 5 Marine Zoology, Bremen Marine Ecology (BreMarE), University of Bremen, PO Box 330440, 28334 Bremen, Germany; 6 Alfred Wegener Institute, Helmholtz Centre for Polar and Marine Research, Am Handelshafen 12, 27570 Bremerhaven; 7 Canadian Museum of Nature, Research and Collections, Station D, Ottawa, Canada; 8 National History Museum, London, Cromwell Road, South Kensington, United Kingdom; 9 University of Bergen, University Museum, Department of Natural History, PO Box 7800, 5020 Bergen, Norway; 10 Tromsø Museum, University of Tromsø, 9037 Tromsø, Norway; 11 Senckenberg am Meer, Department for Marine Biodiversity Research (DZMB), c/o Biocenter Grindel, CeNak: Zoological Museum, Martin-Luther-King-Platz 3, 20146 Hamburg, Germany

**Keywords:** Amphipoda, COI barcoding, deep sea, North Atlantic

## Abstract

Amphipods constitute an abundant part of Icelandic deep-sea zoobenthos yet knowledge of the diversity of this fauna, particularly at the molecular level, is scarce. The present work aims to use molecular methods to investigate genetic variation of the Amphipoda sampled during two IceAGE collecting expeditions. The mitochondrial cytochrome oxidase subunit 1 (COI) of 167 individuals originally assigned to 75 morphospecies was analysed. These targeted morhospecies were readily identifiable by experts using light microscopy and representative of families where there is current ongoing taxonomic research. The study resulted in 81 Barcode Identity Numbers (BINs) (of which >90% were published for the first time), while Automatic Barcode Gap Discovery revealed the existence of 78 to 83 Molecular Operational Taxonomic Units (MOTUs). Six nominal species (*Rhachotropis helleri*, *Arrhis phyllonyx*, *Deflexilodes tenuirostratus*, *Paroediceros propinquus*, *Metopa boeckii*, *Astyra abyssi*) appeared to have a molecular variation higher than the 0.03 threshold of both p-distance and K2P usually used for amphipod species delineation. Conversely, two Oedicerotidae regarded as separate morphospecies clustered together with divergences in the order of intraspecific variation. The incongruence between the BINs associated with presently identified species and the publicly available data of the same taxa was observed in case of *Paramphithoe hystrix* and *Amphilochus manudens*. The findings from this research project highlight the necessity of supporting molecular studies with thorough morphology species analyses.

## Introduction

Within the Class Malacostraca, the Order Amphipoda is currently represented by around 9000 described species, among which 80% are marine (Väinölä et al. 2008). Due to their high diversity and often large abundances (see e.g. Brandt 1997, Brandt et al. 2005, Plaisance et al. 2009), amphipods play a significant role in the food web throughout the worlds oceans (Dauby et al. 2001, 2003).

Studies on the marine zoobenthos around Iceland started in the late 19^th^ Century with the Danish Ingolf Expeditions of 1895 and 1896 (Wandel 1899). These early pioneering cruises included sampling of amphipod fauna and resulted in the published records on amphipod species diversity and distributions (Stephensen 1931, Sæmundsson 1937). In the Century which followed very few articles were produced on the marine amphipods from the Icelandic region. The few papers covered topics of both taxonomy and shallow-water communities (Thurston 1980a, b, Ingólfsson 1996). It was not until the late 1900’s that the Icelandic region received further attention, namely through two large scale research programs, BIOFAR sampling from 1987–1990 (Nørrevang et al. 1994) and BIOICE sampling from 1991–2004 (Brix et al. 2014a). Both these programs were devoted to make an inventory of the marine fauna of the Faroe and Icelandic seas. Successful research continues to be generated from these collections and to-date specific studies of Amphipoda from BIOFAR and BIOICE have included taxonomic works of several families (Larsen 1996, Berge and Vader 1997, Bellan-Santini and Dauvin 1997, Myers 1998, Coleman 1999, Krapp-Schickel 2005, Dauvin et al. 2012), along with zoogeographical and ecological studies which incorporate the abundant and diverse amphipod fauna (Brandt and Piepenburg 1994, Brandt 1997, Weisshappel and Svavarsson 1998, Weisshappel 2000, 2001). Despite the large scale sampling efforts of the BIOFAR and BIOICE programs, it was recognized that large parts of the marine seafloor surrounding Greenland, Iceland and the Norwegian seas were still poorly known. To fill this knowledge gap a research program entitled: Icelandic marine Animals – Genetics and Ecology (IceAGE), was established to further sample and develop our understanding of the North Atlantic marine fauna (Brix et al. 2014a). From the epibenthic sledge samples collected during the IceAGE Expeditions, the Amhipoda are again recognised as an especially abundant and diverse part of the North Atlantic zoobenthos (Brix et al. 2018).

As part of the greater North Atlantic and subarctic region, the special oceanographic conditions associated with the Iceland region and its adjacent waters are particularly interesting (Hansen and Osterhus 2000, Schnurr et al. 2014, Brix et al. 2014a). The marine region around Iceland includes several water masses and a conspicuous submarine mountain chain – the Greenland-Scotland-Ridge (GSR). The ridge topography influences marine habitats and presents a physical barrier separating the Arctic deep-sea basins from the North Atlantic proper. The complex hydrography which occurs across the ridge plays a key role in global thermohaline circulation (Hansen and Osterhus 2000) and is fundamental to the regional Northern European climate. Approaching from the north and engulfing Iceland from both the east and western sides are cold, deep water currents. In contrast, to this deep water encircling, warmer surface waters move around Iceland in a south-west to north-east direction (Ostmann et al. 2014, Schnurr et al. 2014). Although these hydrographical conditions may shape distributions for some isopod groups (Brix and Svavarsson 2010), yet in broader analyses of regional assemblages temperature seen to be less important when compared to other abiotic factors (Schnurr et al. 2014).

Since the proposal of the DNA barcoding concept by Hebert et al. (2003) the use of molecular methods in species recognition has become broadly applied and often supplements morphological taxonomy (e.g. Hubert and Hanner 2015, Seefeldt et al. 2017). The most commonly used molecular marker, is the mitochondrial cytochrome c oxidase subunit 1 (COI) for which there are several protocols available using either universal or specific primers (e.g. Folmer et al. 1994, Geller et al. 2013 and references therein). The use of molecular markers has highlighted the existence of many overlooked species within the Order Amphipoda both in freshwater as well as marine environments (e.g. Lörz et al. 2009, Havermans et al. 2013, Mamos et al. 2016, Verheye et al. 2016). Due to a high diversity and abundance of amphipods within faunal assemblage and the proportionally small number of scientists working on the group, most amphipod studies are restricted to a particular family/species or cover a limited spatial range (see papers cited above). The paper is the first to undertake a broader multi-family and species level approach for studying the molecular diversity of Icelandic amphipods.

Comparative studies on the Icelandic marine fauna have demonstrated a higher than expected molecular diversity for common and widely distributed isopod species (Brix et al. 2014b, Brix et al. in review). A similar pattern may be expected in the case of other peracarid crustaceans, namely the Amphipoda.

The aim of the present study is to use molecular methods to investigate the genetic variation of Icelandic amphipods and understand if changes in molecular diversity reflect the known characteristics of the regional benthic topography and hydrological conditions. The results from this study are a baseline for further research of species diversity and distribution in Icelandic and adjacent waters.

## Material and methods

### Sampling

**Figure 1.**
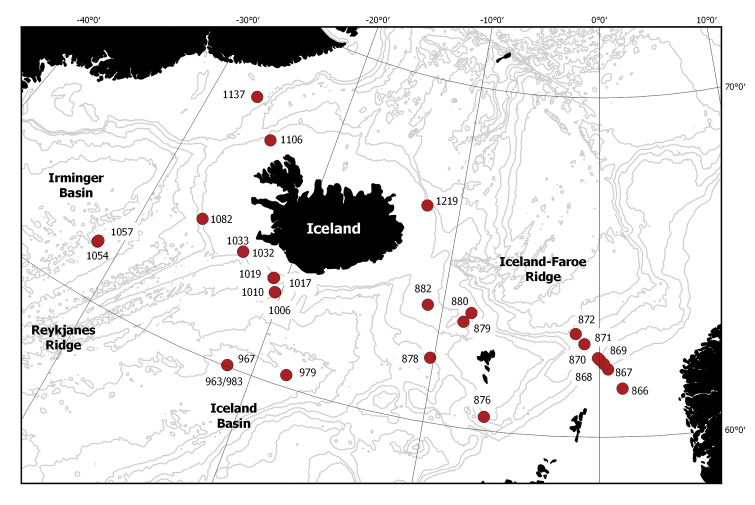
Sampling stations. Depth contours are the following: 500 m, 1000 m, 1500 m, 2000 m, 2500 m, 3000 m. Station details are in Suppl. material 1.

The sampling area covered a wide depth range (from 117 to 2780 m) of the Denmark Strait, Irminger, Iceland and Norwegian basins, as well as the Faroe and Norwegian Channels (Figure 1). Detailed environmental data from each station were also gathered (Brix et al. 2014a).

Samples were taken during IceAGE expeditions 1 and 2 with R/V *Meteor* (M85/3) and R/V *Poseidon* (POS456) in 2011 and 2013 using two types of epibenthic sleds (EBS, Rothlisberg and Pearcy 1977, Brenke 2005). All samples were fixed in precooled (−20°C) 96% undenatured ethanol and treated as described in Riehl et al. (2014).

During two “IceAGE amphipod determination workshops” held at the German Centre for Marine Biodiversity Research (DZMB) in Wilhelmshaven, Germany in July 2016 and in the field station of the University of Lodz in Spała, Poland in May 2017 representatives of recognized families/species were chosen for molecular analysis. Individuals were then determined to species level using Leica (MZ 6, 8 & 12.5) and Nikon (SMZ 800, 1500) dissecting microscopes. World Register of Marine Species (WoRMS) systematic division was followed. Each specimen was separated from the sample and was given a voucher identification number (voucher ID) and will be registered in the ZMH Hamburg. Individuals were subsequently stored at 4°C at the DZMB Hamburg, and DNA extracts are stored at the Smithsonian Institution at −80°C.

One hundred sixty-seven individuals from 27 stations initially assigned by amphipod taxonomists to 75 morphospecies (21 families) were used for molecular analysis (Suppl. material 1). One to six individuals per taxon were chosen. Extraction, PCR and sequencing protocols followed Riehl et al. (2014). Molecular work was conducted by LGC Genomics and the Smithsonian. In the case of individuals from the superfamily Lysianassoidea as well as from families Stegocephalidae and Hyperiopsidae the extraction and PCR protocols of Havermans (2016) were used. For the PCR products, both forward and reverse strands were sequenced using the sequencing services of EUROFINS (Germany).

### Data analyses

Sequences were edited using Geneious 10.1.2 resulting in 167 sequences of length of 621-658 bp excluding primers. All sequences were deposited in GenBank with the accession numbers MG264740-MG264881, KY072917-KY072920 and MG521122-MG521157 (Suppl. material 1). Relevant voucher information, taxonomic classifications, and sequences are accessible through the public data sets “DS-AMPIA” (dx.doi.org/10.5883/DS-AMPIA) and "DS-RHACHOTR" (https://doi.org/10.5883/DS-RHACHOTR) on the Barcode of Life Data Systems (BOLD; www.boldsystems.org) (Ratnasingham and Hebert 2007).

The sequences were aligned with MAFFT v7.308 algorithm with default settings (Katoh et al. 2002, Katoh and Standley 2013) in Geneious 10.1.2 resulting in a 599 bp alignment used for further analyses. Uncorrected p-distance and the Kimura 2-parameter (K2P) model (Kimura1980) were used to determine sequence divergence in MEGA V7.0.18 (Kumar et al. 2016). A Neighbour-Joining (NJ) tree was built based on K2P using the default parameters (transition and transversion substitutions included and pairwise deletion). Node support was inferred with a bootstrap analysis (1000 replicates). The COI sequence of *Pleuroprion hystrix* (G.O. Sars, 1877) (Isopoda) from one of the stations sampled within IceAGE project was used as outgroup.

Two distance-based methods for species delimitation were applied in order to assess the number of MOTUs that could represent putative cryptic species. The first one, Barcode Index Number (BIN) System (Ratnasingham and Hebert 2013), compares newly submitted sequences with the sequences already available in BOLD. They are clustered according to their molecular divergence using algorithms aiming at finding discontinuities between clusters. Each cluster receives a unique and specific code (Barcode Index Number or BIN), either already available or new if submitted sequences do not cluster with already known BINs. The second method, Automatic Barcode Gap Discovery (ABGD) (Puillandre et al. 2012), uses pairwise distance measures. With this method, the sequences are partitioned into groups (MOTUs), such that the distance between two sequences from two different groups will always be larger than a given threshold distance (i.e. barcode gap). One of the critical parameters of the ABGD method is the prior maximum divergence of intraspecific diversity (*P*). The prior *P* values were set from the default value of 0.001 to 0.03. The latter is commonly used for species delimitation in arthropods and particularly in Amphipoda (e.g. Hebert et al. 2003, Costa et al. 2007, 2009, Raupach et al. 2015, Lobo et al. 2017). Both uncorrected p-distance and K2P were used to calculate species distances. Due to a very wide spectrum of taxa used in this study, representing many different families, as well as the presence of large number of singletons our data were not suitable for the phylogenetic approach to species delimitation analysis.

## Results

**Figure 2.**
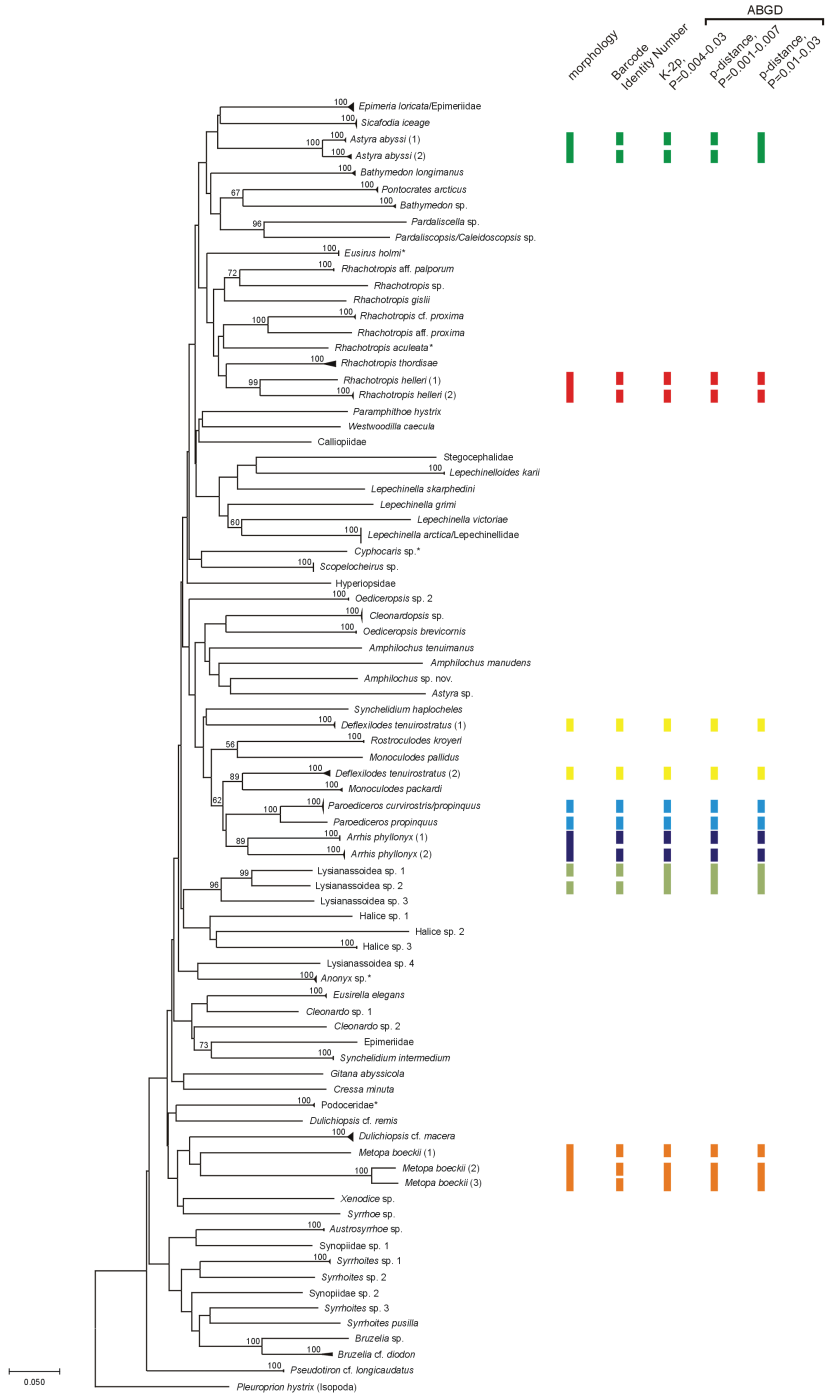
Neighbour-joining (NJ) tree of COI sequences (Suppl. material 1) based on Kimura 2-parameter. Triangles indicate the relative number of individuals studied (height) and sequence divergence (width). The asterisk (*) symbolizes taxa having already published sequences in BOLD/GenBank identified to species level. The numbers in front of the nodes indicate bootstrap support (1000 replicates, only values higher than 50% are presented). The vertical bars represent species delimitations taxonomies obtained from morphology and different species delimitation methods. The same colour indicates the same nominal species. Only the cases where incongruence between different delimitation methods were observed are shown. Note that this tree is not the reconstruction of evolutionary history of presented taxa.

Among the 75 morphologically identified species, 81 Barcode Identity Numbers (BIN) were ascribed by BOLD (Figure 2, Suppl. material 1). Fifty-eight of these are unique for the database, while 23 are shared with other studies. Within the second group, nine are held in private datasets and another nine are left identified at the order level. As a result, only five are public and are associated with known species names. In total, 94% of the BINs in the present study are published for the first time.

The ABGD method allowed for recognition of 79 to 83 MOTUs when using K2P distance and 78–79 MOTUs for p-distance analysis. In the case of K2P the most stable division over a wide range of the prior maximum divergence values (*P*= 0.004-0.03) was 79 MOTUs and thus only this division is presented in Figure 2.

The number of haplotypes for each BIN ranged from one to five, the latter being the case in Dulichiopsis cf. macera (G.O. Sars, 1879) (Table 1). The intraspecific variation expressed by both p-distance and K2P were similar within each BIN and generally low. The highest values were recorded for Bruzelia cf. diodon K.H. Barnard, 1916 and *Rhachotropis thordisae* Thurston, 1980 (0.019 and 0.010, respectively). As many as 43 MOTUs were singletons.

**Table 1.** The intraspecific variation within BINs obtained, calculated using uncorrected p-distance and Kimura 2-parameter (K2P). Taxa represented by a single sequence are not listed.

Family	Taxon	No. of ind.	No. of haplotypes	p-distance	K2P
Amathillopsidae	*Cleonardopsis* sp.	5	2	0.001	0.001
Dulichiidae	Dulichiopsis cf. macera (G.O. Sars, 1879)	6	5	0.005	0.005
Epimeriidae	*Epimeria loricata* G.O. Sars, 1879	6	4	0.006	0.006
Eusiridae	*Eusirella elegans* Chevreux, 1908	2	2	0.002	0.002
Eusiridae	*Eusirus holmi* Hansen, 1887	4	1	0.000	0.000
Eusiridae	Rhachotropis aff. palporum Stebbing, 1908	2	1	0.000	0.000
Eusiridae	Rhachotropis cf. proxima Chevreux, 1911	2	2	0.002	0.002
Eusiridae	*Rhachotropis thordisae* Thurston, 1980	4	2	0.010	0.010
Eusiridae	*Rhachotropis helleri* (2) (Boeck, 1971)	3	2	0.001	0.001
Lepechinellidae	*Lepechinella arctica* Schellenberg, 1926/Lepechinellidae	12	1	0.000	0.000
Lepechinellidae	*Lepechinelloides karii* Thurston, 1980	2	1	0.000	0.000
Oedicerotidae	*Arrhis phyllonyx* (1) M. Sars, 1858	4	1	0.000	0.000
Oedicerotidae	*Arrhis phyllonyx* (2) M. Sars, 1858	4	2	0.001	0.001
Oedicerotidae	*Bathymedon longimanus* (Boeck, 1871)	3	2	0.003	0.003
Oedicerotidae	*Bathymedon* sp.	2	2	0.003	0.003
Oedicerotidae	*Deflexilodes tenuirostratus* (1) (Boeck, 1871)	3	2	0.001	0.001
Oedicerotidae	*Deflexilodes tenuirostratus* (2) (Boeck, 1871)	4	4	0.008	0.008
Oedicerotidae	*Monoculodes packardi* Boeck, 1871	2	2	0.003	0.003
Oedicerotidae	*Oediceropsis brevicornis* (Lilljeborg, 1865)	2	1	0.000	0.000
Oedicerotidae	*Oediceropsis* sp. 2	2	1	0.000	0.000
Oedicerotidae	*Paroediceros curvirostris* (Hansen, 1888)/*P. propinquus* (Goës, 1866)	6	2	0.001	0.001
Oedicerotidae	*Pontocrates arcticus* G.O. Sars, 1895	3	3	0.002	0.002
Oedicerotidae	*Rostroculodes kroyeri* (Boeck, 1870)	2	1	0.000	0.000
Oedicerotidae	*Synchelidium intermedium* (Grube, 1864)	3	1	0.000	0.000
Pardaliscidae	*Halice* sp. 3	2	1	0.000	0.000
Podoceridae	Podoceridae	2	2	0.002	0.002
Scopelocheiridae	*Scopelocheirus* sp.	7	1	0.000	0.000
Sicafodiidae	*Sicafodia iceage* (Campean & Coleman, 2017)	4	2	0.001	0.001
Stilipedidae	*Astyra abyssi* (1) Boeck, 1871	2	2	0.002	0.002
Stilipedidae	*Astyra abyssi* (2) Boeck, 1871	3	3	0.006	0.006
Synopiidae	*Austrosyrrhoe* sp.	2	1	0.000	0.000
Synopiidae	Bruzelia cf. diodon K.H. Barnard, 1925	2	2	0.019	0.019
Synopiidae	Pseudotiron cf. longicaudatus Pirlot, 1934	2	1	0.000	0.000
Synopiidae	*Syrrhoites pusilla* Enequist, 1949	2	2	0.002	0.002
Uristidae	*Anonyx* sp.	4	2	0.002	0.002

Four species identified on the basis of morphology (*Rhachotropis helleri* (Boeck, 1871), *Arrhis phyllonyx* (M. Sars, 1858), *Deflexilodes tenuirostratus* (Boeck, 1871), *Metopa boeckii* G.O. Sars, 1892) showed intraspecific variation considerably exceeding the values commonly used for amphipod species delimitation (Table 2) indicating potential cryptic diversity. For another two species (*Paroediceros propinquus* (Goës, 1866) and *Astyra abyssi* Boeck, 1871) those values were very close to the threshold.

**Table 2.** The values of uncorrected p-distance, Kimura 2-parameter (K2P) and Barcode Identity Numbers (BINs) for nominal species presenting the highest intraspecific variation.

Family	Species	No of ind.	No of haplotypes	p-distance	K2P	BIN
Eusiridae	*Rhachotropis helleri*	4	3	0.076	0.085	ADE3179, ADE4377
Oedicerotidae	*Arrhis phyllonyx*	8	3	0.093	0.106	AAG7255, ADG9371
Oedicerotidae	*Deflexilodes tenuirostratus*	7	6	0.118	0.139	ADH2072, ADH2071
Oedicerotidae	*Paroediceros propinquus*	3	2	0.056	0.060	ADG8965, ACV0335
Stenothoidae	*Metopa boeckii*	3	3	0.198	0.245	ADH5455, ADH5456, ADH5457
Stilipedidae	*Astyra abyssi*	5	5	0.032	0.033	ADG9308, ADG9037

The NJ tree showed the existence of different lineages within the above-mentioned species (Figure 2). Also it revealed that some individuals morphologically identified as *Paroediceros propinquus* have clustered with *Paroediceros curvirostris* (Hansen, 1888). It confirmed also the identity of six individuals originally left identified at the family level (Lepechinellidae) as aligning with specimens identified as *Lepechinella arctica* Schellenberg, 1926.

Incongruence between morphological species identification and different species delimitation methods was observed in the case of two representatives of Lysianassoidea (sp. 1 and sp. 2) (Figure 2). Based on their morphology they were determined as two separate units, which was confirmed by assignation of two different BINs. However, the ABGD method on both p-distance and K2P treated them as a single MOTU. When both sequences were considered together the distance value between them is 0.106 and 0.118 for p-distance and K2P, respectively. In this case the ABGD method seemed to fail, artificially treating two very divergent sequences (and as a result two species) as a single unit.

## Discussion

The present study gives a first “glimpse” into the molecular diversity of Icelandic Amphipoda and provides a baseline for future studies. Further research is needed for where molecular diversity in not congruent with morphological identification. Re-examination of material for characters in consideration of clear alignment of lineages with topology, hydrology and depth stratification is also required. In considering the number of more than 21500 amphipod specimens identified to family level during IceAGE determination workshops (see Brix et al. 2018), only about 170 specimens, 0.7%, of these were selected for barcoding. The specimens targeted for molecular analysis were material identified as in good morphological condition (majority of limbs intact), material which was readily identifiable using light microscopy (did not require dissection and slide preparation for mouth parts), but where largely defined as groups of scientific interest to the experts and where there is current ongoing taxonomic research. The relatively high number of representatives of Eusiridae, Oedicerotidae or Synopiidae reflects the intention of particular scientists to analyse these taxa further. It does not represent the diversity of Icelandic and adjacent waters, as the super abundant and speciose groups such as Phoxocephalidae or Lysianassoidea are acknowledged as underrepresented in this paper. Knowing the limitations associated with the size of the material used for the study it is still possible to define the emerging issues and propose directions for further studies.

### 1. Recognizing amphipod species diversity in Icelandic waters

Based on the material studied 81 BINs were recognized. Only five of the BINs are identified to the species level and publically available, while 94% are either unique, held in private datasets, or without detailed identification. That proportion indicates the extent to which knowledge of this important group of marine zoobenthos is still poorly known. In another barcoding study of Crustacea from Gulf of St. Lawrence (North Atlantic) new barcodes accounted for 75 percent of studied sequences (Radulovici et al. 2009). In the eight years since the release of this earlier study, there is still large gaps in the knowledge of genetic diversity including the deeper parts of the ocean as demonstrated here for the Icelandic and adjacent waters in the North Atlantic. Within the acknowledge limitations of DNA barcoding approach, the present results show that biodiversity studies in Icelandic waters can strongly benefit from the usage of molecular method. According to Ratnasingham and Hebert (2013) the BIN corresponds a distance-based COI sequence cluster that might represent single species. Another species delimitation method (ABGD) revealed the existence of 78 to79 MOTUs (Figure 2). These differences might be explained by methodological difference or alternately by insufficient sampling. The majority of studied taxa were represented by a two or three sequences, which may have prevented proper discrimination between intra and interspecific variation. Based on the present study it is not possible to conclusively assess which of these species delimitation methods gives the most reliable results. The number of individuals per taxon presently studied was low and half of the morphospecies were represented by single sequence only. The most commonly used value for barcode gap was applied here as a threshold to divide species, but there are some works that mentioned higher intraspecific diversity within deep-sea amphipods than previously expected (Knox et al. 2012).

The present study allowed for obtaining barcodes for species newly described from Icelandic waters: *Sicafodia iceage* Campean & Coleman, 2017 and *Amphilochus anoculus* Tandberg & Vader, 2018 (Campean and Coleman 2017, Tandberg and Vader 2018). Additionally, based on the combination of morphological and molecular data some species belonging to the genera *Rhachotropis, Bruzelia*, *Austrosyrrhoe* and *Syrrhoites* have been recognized as putatively new to science.

It is important to point out that the taxonomic and molecular diversity that can be seen in the NJ tree does not reflect the complete amphipod family and species diversity of Icelandic and adjacent waters, but reflects only a small representation, less than 1% of processed samples, were investigated here for genetic analysis.

### 2. Morphological versus molecular species identification

The molecular results are generally congruent with the morphological identification of studied species. The existence of potential cryptic (or pseudocryptic) species has been observed within three taxa of Oedicerotidae as well as one taxon in the families: Eusiridae, Stilipedidae and Stenothoidae.

Two clearly distinct clades have been observed within *Rhachotropis helleri* (Eusiridae). The specimens representing both lineages were collected at similar depths (ca. 300 m) but from very different localities: the Iceland-Faroe Ridge and the Iceland Basin. As the genus *Rhachotropis* is the subject of another publication in this issue (Lörz et al. 2018) the details of taxonomic rank of *R. helleri* are not presented here.

In *Arrhis phyllonyx* (Oedicerotidae) two different lineages have been recognised for this study. *Arrhis phyllonyx* is a species commonly reported from North Atlantic waters with a wide depth range from 100 to 2680 m (Sars 1890, Vader unpublished data). Some morphological variability has been observed and might be associated with the depth distribution of this taxon. Morphological studies have previously documented the subspecies—*A. phyllonyx arcticus* Bryazgin, 1974—from the Barents Sea (Bryazgin 1974). In the present study, all specimens were collected in the Iceland-Faroe Ridge area at neighbouring stations, including 510 m depth (lineage 1) and 158 to 686 m depth (lineage 2). Further detailed study of the morphology variation along with molecular analyses is required.

Two different clades of *Deflexilodes tenuirostratus* have been observed where genetic separation aligns with difference in sampling locality, with clade 1 sampled from the Iceland Basin and clade 2 sampled from the Iceland-Faroe Ridge. Given the clear geographic distinction between clades additional research is required to more closely investigate the morphology to assess if there could exist two cryptic species within this taxon.

Smaller yet consistent sequence differences were also noted in *Paroediceros propinquus*. All individuals sequenced were collected from similar depths at neighbouring stations on the Iceland-Faroe Ridge. Moreover, the sequences of *P. propinquus* forming clade 1 appeared to share haplotypes with another species in this genus, namely *P. curvirostris* indicating that the morphological characters require closer examination to see if these BINs can be supported with additional morphological character states.

The results for the family Oedicerotidae will be further studied using additional genes and material from other localities (Hughes pers. com.). It is worth noting that similar results were recently observed in the case of some other North Atlantic amphipod species reported as having wide distribution range for six out of the 68 identified morphospecies (Lobo et al. 2017). In their case study the incongruence between the morphological identification and genetic variability was explained by geographic distance in four of the disparate morphospecies. In the remaining two amphipods, *Corophium multisetosum* Stock, 1952 and *Dexamine spiniventris* (Costa, 1853) the species presented high genetic divergence were collected in the same area. A lack of morphological characters differentiating two sympatrically distributed lineages of a single recognised morphospecies was observed also in *Leucothoe vulgaris* White & Reimer, 2012 (White et al. 2015). With morphologically conservative yet genetically defined species appearing across amphipod families the disparate results from these methods prompt more fine scale morphological and broader molecular investigation.

High genetic diversity was also observed in one species from the family Stenothoidae: *Metopa boeckii*. Depending on the species delimitation method, two (ABGD) or three (BINs) MOTUs have been revealed. Some morphological variability within this species has already been observed and further morphological studies could result in new species description. All individuals of this nominal species were collected in the same geographic area at similar depths, but on opposite sides of Iceland-Faroe Ridge: *M. boeckii* lineage 1 occurred south of the topographic barrier while lineages 2 and 3 were collected from the north side. The representatives of Stenothoidae are often known to occur in association with other invertebrates (Brix et al. 2018). A further examination of host data could reveal if its dispersal limitation is potentially defined by the host invertebrate. The Island-Faroe Ridge has been demonstrated as a defining feature to dispersal for the North Atlantic isopods from the genus *Oecidiobranchus* (Brix et al. in review).

With the family Stilipedidae delimiting the species *Astyra abyssi* can be seen as either one or two species depending on the methodology applied. The values of p-distance and K2P are just over the threshold that is commonly used to discriminate species of arthropods and amphipods in particular (Hebert et al. 2003, Costa et al. 2007, 2009). *Astyra abyssi* was represented by five individuals in this study, and further molecular analysis of individuals would be needed to confirm if the observed diversity represents high intraspecific variation or of the presence of two species, one of which is cryptic. The two lineages are seen to be depth stratified with *A. abyssi* lineage 1 (2 individuals) occurred at ~300 m south of Iceland, while the lineage 2 are from two deeper water station of 724 m and 1385 m, respectively in the Irminger and Iceland basins.

These two deeper water stations, the Irminger and Iceland basins, are separated by the Reykjanes Ridge, a topological feature. However, these separated locations could be connected by the movement of water masses around Iceland, as this pattern is also seen in other deep-sea peracarids (Svavarsson et al. 1993; Svavarsson 1997, Negoescu and Svavarsson 1997). The lack of genetic separation of the populations collected from both sides of Reykjanes Ridge are also known for the isopod *Chelator insignis* (Hansen, 1916) species complex (Brix et al. 2014b). Both deep-water stations are situated in areas influenced by deep, cold currents flowing from the northeast and passing by the Reykjanes Ridge (Ostmann et al. 2014). The representatives of the family Stilipedidae are regarded as having good swimming abilities, with some species considered as pelagic (Berge 2003). By contrast, *Astyra abyssi* in lineage 1 is from a more shallow water station with an area of warm surface water current (Ostmann et al. 2014). The influence of vertical distribution on genetic divergence is known for the deep sea amphipod *Eurythenes gryllus* (Lichtenstein in Mandt, 1822) with clear separation between lineages inhabiting bathyal and abyssal depths (Havermans et al. 2013; Havermans 2016). The separation of lineages associated with depth and related to different water masses was observed in the case of pelagic siphonophore species in Sagami Bay, Japan (Grossmann et al. 2013) where two molecularly distinct populations of *Lensia achilles* Totton, 1941 were correlated with warm subtropical and cold subarctic water masses.

The present study assisted with delimiting specimens suspected to be juvenile forms to be evaluated to a species level along side congeneric BINs. Several juvenile lepechinellids initially identified only to the family level (Lepechinellidae) were able to be assigned to *Lepechinella arctica*. In this way molecular analyses was useful where ontogenic stage restricts morphological identification of individuals.

### 3. Comparison of IceAGE barcodes with publicly available content

Molecular methods proved to be a useful tool in cryptic species recognition, and the existence of several amphipod species complexes has been already reported (Lörz et al. 2009, Havermans et al. 2013, Mamos et al. 2016, Verheye et al. 2016). Species initially treated as taxa with wide geographic distributions are often review following genetic analyses, especially where genetic lineages show distributions divergence in association with topography, hydrology or depth. The existence of a species complex was observed in *Paramphithoe hystrix* (Ross, 1835) (Schnabel and Hebert 2003). In the present study the *P. hystrix* sequence obtained was recognized as a unique BIN for BOLD, therefore this study contributes another lineage to this known *P. hystrix* complex. At present the complex is not supported by a morphological assessment which would allow comparison of the voucher specimens. Vouchered taxonomic identifications are essential for genetic studies, as once the mistake appear in barcoding database it is easily repeated by further users of the online genetic resources. Without currently published information on the morphology associated with these lineages, at present there can be no further comparison of this species complex as MOTUs (BINs) with the morphological concept of *P. hystrix* in the taxonomic literature.

For *Amphilochus manudens* Spence Bate, 1862 it appears also, that the individual which was assigned to this species from IceAGE sampling represents a different BIN than the specimens collected from the North Sea and ascribed to the same taxon. The sequence divergence is large (0.228 p-distance and 0.278 K2P) much higher than the present concept for intraspecific variation. The two MOTUs observed within the nominal *A. manudens* have different geographic and bathymetric distributions. The specimen from IceAGE was collected in the area of the Iceland-Faroe Ridge at 500 m depth, while the previously reported material came from a shallow station (50 m) in southeast North Sea (Raupach et al. 2015). Further studies of voucher material should be conducted to assess the comparative morphology of the material and possibly that of type material.

## Conclusion

DNA barcoding can help considerably in recognition of species diversity in the deep sea by indicating the existence of cryptic or pseudocryptic species and allowing the taxonomists to focus on the novel morphological and genetic incongruence. However, the accuracy of the taxonomic identification of records in molecular databases is crucial to make those databases reliable for further users. The current study of Amphipoda from Icelandic and adjacent water in the North Atlantic strongly recognises that molecular methods need to be supplemented by comprehensive taxonomical analysis of species morphology in order to provide an expert certified baseline for further biodiversity studies.

## Supplementary materials

Supplementary material 1 Table S1 Data type: molecular data Explanation note: Amphipod and outgroup accession numbers in BOLD, GenBank and station data. This is an open access article distributed under the terms of the Creative Commons Attribution License (CC BY 4.0), which permits unrestricted use, distribution, and reproduction in any medium, provided the original author and source are credited.Anna M. Jażdżewska, Laure Corbari, Amy Driskell, Inmaculada Frutos, Charlotte Havermans, Ed Hendrycks, Lauren Hughes, Anne-Nina Lörz, Bente Stransky, Anne Helene S. Tandberg, Wim Vader, Saskia Brix
